# Association of Subjective Global Assessment with outcomes in the intensive care unit: A retrospective cohort study

**DOI:** 10.1111/1747-0080.12767

**Published:** 2022-09-20

**Authors:** Suzie Ferrie, Nina Bianca Weiss, Hiu Yi Chau, Sophia Torkel, Morgan Elizabeth Stepniewski

**Affiliations:** ^1^ Department of Nutrition & Dietetics Royal Prince Alfred Hospital Camperdown New South Wales Australia; ^2^ Faculty of Medicine and Health University of Sydney Sydney Australia

**Keywords:** critical illness, length of stay, malnutrition, mortality, nutrition assessment

## Abstract

**Aims:**

This retrospective audit was conducted to investigate the association between outcome and protein‐energy malnutrition diagnosed using Subjective Global Assessment (SGA), to evaluate the predictive validity of Subjective Global Assessment in adults admitted to intensive care.

**Methods:**

The audit analysed the medical records of 1034 consecutive adult patients who had nutrition assessment on admission to the intensive care unit between January 2017 and July 2018. Extracted data included patient demographics, nutritional status, outcomes, and Acute Physiology and Chronic Health Evaluation II score. Regression was used to explore the association between Subjective Global Assessment and outcomes.

**Results:**

The prevalence of protein‐energy malnutrition was 39.5% (342 patients SGA‐B, and 75 patients SGA‐C), and there was a significant independent association between Subjective Global Assessment and outcomes both in surgical and non‐surgical patients. Compared with well‐nourished patients, mortality was significantly higher in the malnourished, during the intensive care admission (*p* = 0.007), in hospital (*p* < 0.0001), at 90 days (*p* = 0.001) and at 180 days (*p* = 0.002). Pressure injuries were more common (*p* = 0.01). Length of stay was longer in intensive care (*p* = 0.001) and in hospital (*p* < 0.001), with increased readmission rate (*p* < 0.001).

**Conclusion:**

Protein‐energy malnutrition diagnosed by Subjective Global Assessment had a significant independent association with adverse clinical outcomes in critically ill patients. Subjective Global Assessment appears to have predictive validity in this patient population.

## 1 INTRODUCTION

Malnutrition is known to increase the risk of adverse patient outcomes including mortality, morbidity and length of stay in the acute hospital setting.^1^, ^2^ Malnutrition is common in hospital patients in Australia^1^, ^3^, ^4^ and worldwide.^2^ Amongst hospitalised patients, undernutrition (or inadequate nutrient consumption) is combined with disease‐related metabolic alterations that promote catabolism, attributable to acute or chronic inflammation.^1^, ^5^ The effect on outcomes is due to decline in functional capacity as a result of muscle and fat losses,^6^ reduced respiratory and cardiac function, impaired immune function,^7^ and a diminished capacity for intestinal absorption.^8^ Protein‐energy malnutrition may also contribute to fatigue and apathy in patients, ultimately diminishing quality of life. These effects would be expected to apply to all hospital patients including the critically ill who are at even higher risk of lean tissue losses due to the nature of acute illness.^9^

Identifying malnutrition requires a nutritional assessment, and there are several established standardised tools for this purpose. One widely‐used example is Subjective Global Assessment (SGA), which classifies nutritional status based on a physical examination and a targeted patient history to gather information about recent change in weight, intake and function, and the presence of nutrition‐impact symptoms.^10^ Originally developed to assess nutritional status and predict clinical outcomes in surgical patients, the SGA is regarded by many clinicians as the gold standard method for diagnosing protein‐energy malnutrition due to its simplicity and reproducibility; it has been validated against alternative measures of nutritional status^11^ and as a predictor of outcomes in a variety of clinical areas such as in rehabilitation and geriatrics,^12^, ^13^ renal,^14^ liver transplant,^15^ and oncology patients.^16^ It does have some limitations: the subjective nature of the assessment can result in variable inter‐rater reliability^17^ and, having only three categories, the lack of sensitivity to short‐term changes means SGA is not suitable for day‐to‐day monitoring.^18^ Within its intended use as an assessment tool, it incorporates risk factors for altered tissue metabolism and muscle function as well as reduced muscle mass, making it a more holistic and patient‐focused measure of nutritional status than ‘objective’ measures of body composition can be. In the ICU, however, it is still common to find statements in the literature about its lack of validity^19^, ^20^ or clinicians raising concerns about its feasibility in the ICU setting when history cannot be obtained from unconscious patients^21^ despite the fact that obtaining corroborating history from the patient's family was actually suggested by the SGA's original authors as part of its valid methodology.^22^ Despite these expressed doubts, SGA is the most common assessment method used by ICU dietitians in Australia and New Zealand.^23^ Its subjective, inferential approach may in fact be helpful in the ICU setting^24^ given that objective measurements including anthropometric and biochemical markers may not be reliable as they can be affected by several clinical factors, such as fluid retention and acute phase response which are common during critical illness.^25^ In terms of validity, a number of previous studies suggest that SGA has predictive validity with associations between SGA and various ICU outcomes, but many of these studies were small^26^, ^27^, ^28^ or confounded by a failure to adjust for other important outcome influences such as severity of illness^29^, ^30^, ^31^ and this may be why questions remain about the use of SGA in ICU.

The aim of the current study was to examine the predictive validity of SGA in adults admitted to ICU, using a retrospective medical record audit to evaluate the association between SGA and outcomes including mortality, length of stay, time on ventilator, hospital readmission, use of antibiotics, and incidence of pressure injury or positive blood culture, including a large number of patients, and adjusting for relevant confounders such as severity of illness.

## 2 METHODS

The study was conducted in the mixed medical/surgical ICU of a large metropolitan tertiary‐referral teaching hospital. The retrospective audit included 1034 consecutive adult patients who had been assessed by a dietitian using SGA on admission to the ICU between January 2017 and July 2018. The electronic medical records of these patients were obtained to extract data on patient demographics, nutritional status, outcomes, and Acute Physiology and Chronic Health Evaluation II (APACHE II)^32^ score which was used as the measure of severity of illness. Where a patient was readmitted to the ICU within a single hospital admission, only the first ICU admission with a documented SGA was included. The patient's APACHE II score was calculated from the first 24 h of that same ICU admission. The study was approved by the hospital's ethical review committee (approval X19‐0059 & 2019/ETH00428, 21 May 2019) and reporting followed the STROBE‐nut guidelines.^33^

**FIGURE 1:**
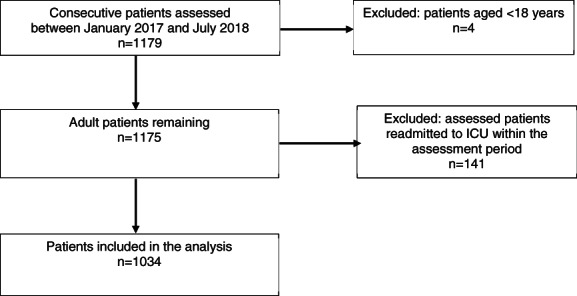
Study flowsheet for Intensive Care Unit patients included in the audit

Consecutive patients were included if there was an SGA score documented by the intensive care dietitian within the first 72 h of ICU admission. Patients were excluded if they were aged <18 years or were readmitted to ICU within the same hospital admission where the previous ICU admission had been included in the audit, see Figure 1 for study flowsheet.

Nutritional status was assessed using SGA upon patient admission to the ICU. The procedure in this ICU is for nursing staff to screen every patient on admission to ICU and then regularly thereafter, using the Malnutrition Screening Tool,^34^ and an automatic dietitian referral is made if the tool indicates malnutrition risk (a score of 2 or more). The dietitian is also referred if the patient requires nutrition support or if there are any other nutritional concerns, and SGA is a mandatory part of the initial assessment for all patients referred to the dietitian. For all patients included in the study, the SGA was performed by one of two experienced intensive care dietitians. To complete the assessment, alongside a physical examination, information about previous weight loss, changes in dietary intake, nutrition impact symptoms and functional capacity were collected from the patient where possible, or from a family member and/or patient medical records. The subjective summation of all the information resulted in a score of acceptably nourished (SGA‐A), mild to moderately malnourished (SGA‐B) or severely malnourished (SGA‐C). To reduce variation, agreement on SGA classification was regularly calibrated between the two dietitians at quarterly clinical practice consensus reviews.

To reduce bias, outcomes were extracted from each patient's electronic medical record by researchers who were blinded to the patient's SGA classification. Random patients throughout the dataset had their data checked by a second independent researcher. Length of stay in ICU and in hospital were automatically calculated as 24‐hour days within the medical record. To remove the competing risk of mortality which can contribute to survivor bias, patients who died in ICU were excluded from the ICU length‐of‐stay analysis, and similarly patients who died in hospital were excluded from the hospital length‐of‐stay. Patient mortality status at 90 and 180 days after ICU admission was also determined from patient medical records. Patients without a deceased status shown in the electronic medical record were assumed to be alive. Length of time on mechanical ventilation, recorded in days, was included in the analysis, but additionally to remove the competing risk of mortality, analyses were performed in survivors only, as well as using the length of time on mechanical ventilation converted to the number of days alive free of ventilation within the first 28 days after ICU admission. Competing‐risks regression was also conducted to explore the association between SGA and time on mechanical ventilator. Patients were counted as receiving antibiotics if they received at least one dose of any antimicrobial medication, excluding those administered solely as part of a surgical prophylaxis protocol. Patients were counted as intolerant to enteral nutrition if feeds were significantly interrupted due to gastrointestinal signs or symptoms including abdominal distension or discomfort, vomiting or increased gastric aspirates, resulting in a delay of more than 24 h to commence enteral nutrition or more than 3 days to reach goal feed rate after ICU admission.

For the reporting and simple analyses, patients were divided into three groups according to the SGA score. Study size was determined by the available resources for data collection. Categorical variables were presented as frequency (%). Continuous variables were presented as mean ± standard deviation for normally distributed data, otherwise median (inter‐quartile range) was used. Competing‐risks regression analysis was performed using SAS Studio 3.8 (SAS Institute Inc, Cary, NC). All other statistical analyses were performed using SPSS Statistics 26.0 (IBM Corp, Armonk, NY). There were no missing data.

Predictive validity of SGA was assessed by exploring the associations between malnutrition classified by SGA, and clinical outcomes, using standard multiple linear and logistic regression analyses to adjust for age, sex, and severity of illness as measured by the APACHE II score. The outcomes investigated were mortality, length of stay, readmission, ventilator days, incidence of positive blood culture, use of antibiotics, incidence of pressure injury, primary route of nutrition support, and intolerance to enteral nutrition. For the regression analyses, preliminary analysis was conducted to ensure no violation of the assumptions of normality, linearity, multi‐collinearity and homoscedasticity. The normality of continuous variables was evaluated using the Kolmogorov–Smirnov test. SGA category was dichotomised as acceptably‐nourished (SGA‐A) versus malnourished (SGA‐B + SGA‐C). The potential covariates with significant bivariate correlation with the dependent variable were included in the model if collinearity was not identified. Collinearity was assessed using Spearman's rank correlation coefficient and defined as rho ≥0.7. A separate logistic regression was conducted for each of the outcomes of interest (dependent variables) that were dichotomous: mortality at different timepoints (coded as yes or no), antibiotics (yes or no), positive blood culture (yes or no), pressure injury (yes or no), main route of nutrition (enteral nutrition or parenteral nutrition), and intolerance to enteral nutrition (yes or no). A standard multiple regression was conducted for each of the outcomes that were continuous variables: length of stay (days) and time on mechanical ventilation (days). The covariates included in all analyses were: age (years), sex (coded as male or female), APACHE II (points). Overfitting of the model was avoided with no more than three independent covariates included. Goodness of fit was examined using the Hosmer‐Lemeshow goodness of fit test and poor fit was defined as *p* < 0.05.

Nine outliers were removed from the regression analysis after being identified by very large Mahalanobis Distance. Across the three independent SGA groups, continuous variables were compared using Welch's Analysis of Variance or Kruskal‐Wallis test, and Chi‐square test was used for categorical variables. A *p* value <0.05 was considered statistically significant.

## 3 RESULTS

A total of 1034 patient medical records were reviewed in this audit. Patient demographics are shown in Table 1. More patients were male (60.9%) and admitted as surgical ICU patients (59.4%). Men were older with a mean age of 60.63 years (SD 15.04), compared to women whose mean age was 57.12 years (SD 17.30), *p* = 0.0006. At ICU admission, 327 (38.5%) patients were classified as malnourished. Notably, more women than men were malnourished, *p* = 0.039. ICU nutrition protocols were followed, with nutrition support commenced within 24 h of ICU admission in 76% of patients, overall average 16.2 h after arriving in ICU, reaching 100% goal rate average 2.4 days after arriving in ICU.

**TABLE 1:** Demographic characteristics of patients at time of Intensive Care Unit admission

Demographic characteristic	
Age, years, mean (SD)	59.26 (16.05)
Sex, *n* (%)	
Male	630 (60.9)
Female	404 (39.1)
APACHE II score, mean (SD)	19.93 (7.05)
SGA score, *n* (%)	
A	636 (61.5)
B	327 (31.6)
C	71 (6.9)
Main reason for ICU admission, n (%)	
Surgical	614 (59.4)
Gastrointestinal/abdominal surgical	286 (27.7)
Cardiothoracic surgical	98 (9.5)
Neurological surgical	97 (9.4)
Liver surgical	86 (8.3)
Trauma/orthopaedic surgical	27 (2.6)
Other surgical	20 (1.9)
Medical	420 (40.6)
Respiratory	82 (7.9)
Cardiac medical	77 (7.4)
Neurological medical	75 (7.3)
Gastrointestinal/abdominal medical	56 (5.4)
Sepsis	45 (4.4)
Haematology	26 (2.5)
Liver medical	25 (2.4)
Renal medical	14 (1.4)
Other medical	20 (1.9)
ICU length of stay, days, median (IQR)	7.00 (7.00)
Hospital length of stay, days, median (IQR)	23.00 (24.00)
Days receiving mechanical ventilation, median (IQR)	3.00 (6.00)
Non‐ventilated days alive in first 28 days, days, median (IQR)	25.00 (11.00)
Died in ICU, *n* (%)	159 (15.4%)
Died in hospital, *n* (%)	219 (21.2%)

Abbreviations: APACHE, acute physiology and chronic health evaluation score; ICU, intensive care unit; IQR, inter‐quartile range; SD, standard deviation; SGA, subjective global assessment (SGA‐A acceptably‐nourished, SGA‐B mild‐to‐moderately malnourished, SGA‐C severely malnourished).

Table 2 shows the patient demographics and clinical outcomes stratified by SGA score. APACHE II score, age and sex all had a significant independent association with most of these outcomes. After adjusting for these parameters, there was a significant independent influence of nutritional status as diagnosed using SGA, on a variety of clinical outcomes in these ICU patients, see Table 3. The results were very similar when analysed separately in surgical patients or non‐surgical patients. Overall, severely malnourished patients were significantly more likely to die in ICU (SGA‐C vs. SGA‐A, *p* = 0.007) or in hospital (SGA‐C vs. SGA‐A, *p* < 0.001) and had significantly higher 90‐day mortality (SGA‐C vs. SGA‐A, *p* = 0.002) and 180‐day mortality (SGA‐C vs. SGA‐A, *p* = 0.002). Of those patients who survived to ICU discharge, ICU length of stay was significantly longer for the malnourished (F[21064] = 12.86, *p* < 0.001), with an R^2^ of 0.024, that is, 1.86 days were added to ICU admission for each increase in SGA severity of malnutrition category. Both SGA and APACHE II score were significant independent predictors of ICU length of stay in ICU survivors. Of those patients who survived to hospital discharge, length of stay in hospital was significantly longer for the malnourished (F[21064] = 11.01, *p* < 0.001), with an R^2^ of 0.02 with a median length of stay 27 days in the malnourished patients (SGA‐B/C) compared with 21 days in the SGA‐A group. Both SGA and APACHE II score were significant independent predictors of hospital length of stay in hospital survivors. Malnourished patients were more likely to be readmitted to hospital within 90 days (*p* < 0.0001) or 180 days (*p* < 0.0001) and were more likely to have a documented pressure injury during the admission (*p* = 0.004).

**TABLE 2:** Patient demographics and outcomes according to Subjective Global Assessment classification

Demographic characteristic	SGA Score		
	A	B	C
Age, years, mean (SD)	58.49 (16.71)	60.75 (14.64)	59.28 (15.94)
Sex, male, *n* (%)	403/636 (63.4%)	190/327 (58.1%)	37/71 (52.1%)
APACHE II score, mean (SD)	19.57 (7.27)	20.48 (6.76)	20.56 (6.21)
Outcome			
Died in ICU, *n* (%)	97/636 (15.3%)	42/327 (12.8%)	20/71 (28.2%)
Died in hospital, *n* (%)	127/636 (20.0%)	65/327 (19.9%)	27/71 (38.0%)
90‐day mortality, *n* (%)	137/636 (21.5%)	66/327 (20.2%)	27/71 (38.0%)
180‐day mortality, *n* (%)	142/636 (22.3%)	76/327 (23.2%)	28/71 (39.4%)
Readmitted within 90 days, *n* (%)	111/636 (17.5%)	90/327 (27.5%)	15/71 (21.1%)
Readmitted within 180 days, *n* (%)	165/636 (25.9%)	123/327 (37.6%)	22/71 (31.0%)
ICU length of stay, days, median (IQR)	7.00 (8.00)	6.00 (6.00)	7.00 (6.00)
Hospital length of stay, days, median (IQR)	21.00 (21.00)	26.00 (30.00)	34.00 (30.00)
Days receiving mechanical ventilation, median (IQR)	3.00 (7.00)	2.00 (5.00)	1.50 (4.00)
Non‐ventilated days alive in first 28 days, days, median (IQR)	24.00 (12.00)	26.00 (7.00)	26.00 (15.00)
Positive blood culture, *n* (%)	124/636 (19.5%)	65/327 (19.9%)	14/71 (19.7%)
Use of antibiotics in ICU, *n* (%)	351/636 (55.2%)	166/327 (50.8%)	39/71 (54.9%)
Received EN as main source of nutrition, *n* (%)	429/636 (67.5%)	188/327 (57.5%)	34/71 (47.9%)
Pressure injury, *n* (%)	79/636 (12.4%)	42/327 (12.8%)	17/71 (23.9%)
Intolerance to enteral nutrition^a^	57/429 (13.3%)	19/188 (10.1%)	4/34 (11.8%)

Abbreviations: APACHE, acute physiology and chronic health evaluation score; EN, enteral nutrition; ICU, intensive care unit; IQR, inter‐quartile range; SD, standard deviation; SGA, subjective global assessment (SGA‐A acceptably‐nourished, SGA‐B mild‐to‐moderately malnourished, SGA‐C severely malnourished).

^a^ Intolerance to enteral nutrition defined as any gastrointestinal signs or symptoms (abdominal distension or discomfort, increased gastric aspirates, vomiting) significantly delaying/interrupting EN delivery.

**TABLE 3:** Association between Subjective Global Assessment classification and Intensive Care Unit outcomes: regression results (adjusted for severity of illness^a^)

	Logistic regression analysis (comparing to SGA‐A)			
Variables			Adjusted odds ratio (95% CI)	*p*
ICU mortality		SGA‐B SGA‐C	0.78 (0.52, 1.16) 2.20 (1.23, 3.91)	0.22 0.007
Hospital mortality		SGA‐B SGA‐C	0.94 (0.66, 1.33) 2.59 (1.52, 4.41)	0.71 <0.001
90‐day mortality		SGA‐B SGA‐C	0.86 (0.61, 1.21) 2.29 (1.35, 3.89)	0.39 0.002
180‐day mortality		SGA‐B SGA‐C	0.98 (0.70, 1.37) 2.27 (1.35, 3.85)	0.90 0.002
Readmitted within 90 days (survivors)		SGA‐B SGA‐C	1.78 (1.30, 2.43) 1.24 (0.69, 2.23)	<0.001 0.47
Readmitted within 180 days (survivors)		SGA‐B SGA‐C	1.74 (1.31, 2.30) 1.29 (0.77, 2.16)	<0.001 0.33
Incidence of positive blood culture		SGA‐B SGA‐C	1.05 (0.75, 1.45) 1.06 (0.59, 1.91)	0.79 0.84
Use of antibiotics in ICU		SGA‐B SGA‐C	0.84 (0.64, 1.10) 1.02 (0.62, 1.67)	0.21 0.95
Incidence of pressure injury		SGA‐B SGA‐C	1.09 (0.74, 1.60) 2.12 (1.19, 3.79)	0.67 0.01
Intolerance to enteral nutrition^b^		SGA‐B SGA‐C	0.70 (0.41, 1.22) 0.82 (0.23, 2.43)	0.21 0.72

	Multivariate linear regression analysis				
Variables	R^2^		β	B (95% CI)	*p*
ICU length of stay (survivors)	0.024	SGA APACHE II	−0.10 0.12	−1.86 (−3.00, −0.73) 0.21 (0.11, 0.31)	0.001 <0.001
Hospital length of stay (survivors)	0.020	SGA APACHE II	0.13 0.06	9.79 (5.14, 14.44) 0.42 (0.00, 0.83)	<0.001 0.049
Days receiving mechanical ventilation (survivors)	0.070	SGA APACHE II	−0.19 0.21	−1.9 (−2.61, −1.13) 0.19 (0.12, 0.25)	<0.001 <0.001
Non‐ventilated days alive in first 28 days	0.12	SGA APACHE II	0.09 −0.34	1.39 (0.53, 2.26) −0.46 (−0.54, −0.38)	0.002 <0.001

Abbreviations: APACHE, acute physiology and chronic health evaluation score; ICU, intensive care unit; IQR, inter‐quartile range; SD, standard deviation; SGA, subjective global assessment (SGA‐A acceptably‐nourished, SGA‐B mild‐to‐moderately malnourished, SGA‐C severely malnourished).

^a^ Severity of illness was determined by the APACHE II score.

^b^ Intolerance to enteral nutrition defined as any gastrointestinal signs or symptoms (abdominal distension or discomfort, increased gastric aspirates, vomiting) significantly delaying/interrupting feed delivery.

The number of days requiring ventilation was notably lower for malnourished patients, median 2.0 days for SGA‐B and 1.5 days for SGA‐C, compared with 3.0 days for SGA‐A. This was significant (F[2663] = 26.02, *p* < 0.001), with an R^2^ of 0.07. Both SGA and APACHE II score were significant independent predictors of ventilation days. This difference remained significant (*p* < 0.0001) when competing‐risks regression was used to adjust for significant covariates; age, APACHE II score and the competing risk of mortality. Higher APACHE II score was significantly associated with more days of mechanical ventilation, but increasing age was associated with fewer ventilation days.

A slightly higher proportion of severely‐malnourished patients (SGA‐C) received antibiotics in ICU and/or had a documented positive blood culture, but these differences were not significant. APACHE II score, age and sex were all independent significant predictors of these outcomes. Intolerance to enteral feeds was highly associated with age (*p* = 0.01) and with APACHE II score (*p* = 0.024).

## 4 DISCUSSION

In this audit, over one‐third of the patients were assessed as malnourished using SGA, a prevalence of protein‐energy malnutrition of 38.5% in this mixed population of ICU patients with a mean APACHE II score of 20. Comparable results have recently been reported with SGA in a surgical ICU in Singapore where 40% were malnourished (Chua et al.)^35^; in a Canadian medical ICU where 35% of patients were malnourished and the mean APACHE II score was 21 (Bector et al.),^29^ and in a mixed ICU in Turkey where 37% of patients were malnourished and mean APACHE II score was 25 (Sungertekin et al.).^36^ Higher malnutrition rates have been reported recently in a range of ICUs, with 55% of patients malnourished in a medical ICU in India (Verghese et al.)^37^ and comparable rates reported at two city hospitals in Brazil, by Fontes et al. (54% malnourished)^38^ and by Gatterman Pereira et al. (60.5% malnourished).^39^ Notably, in both of these latter studies, the majority of patients had elective surgery, were not ventilated >24 h, and APACHE II scores averaged significantly lower, median 15 and mean 11 respectively, illustrating the important consideration that ICU populations vary widely.

In the present study, after adjusting for age, sex and severity of illness (APACHE II score), there was a significant independent association between SGA and outcome including mortality at a number of different timepoints, length of stay in ICU and in hospital, hospital readmission rate, and incidence of pressure injuries. The findings of this study largely accord with those from previous smaller studies investigating the association between outcomes and protein‐energy malnutrition according to SGA in an ICU setting. Interestingly, there was no clear dose–response relationship for most of the outcomes (increase in risk between SGA‐A and SGA‐B and SGA‐C), possibly because of the small number of patients classified as SGA‐C. Unexpectedly, despite having poorer outcomes otherwise, malnourished patients had fewer days of mechanical ventilation, as did older patients. ‘Days of mechanical ventilation’ as an outcome variable is difficult to analyse due to a number of competing risks.^41^ For example, a shorter length of time requiring mechanical ventilation is a positive outcome if the time was shortened by faster recovery, better respiratory function and earlier extubation, but not if the time was shortened by the death of the patient (competing risk of mortality). How to manage this competing risk in analysis remains controversial. In this study, whether the analysis used competing‐risks regression with the full dataset, or whether only survivors were included in the analysis, or whether a composite measure such as ‘alive non‐ventilated days up to day 28’ was used, malnourished and older patients still had significantly shorter ventilation time. This unexpected result may reflect a tendency towards earlier withdrawal of mechanical ventilation, and the use of a wider variety of non‐mechanical ventilation strategies, in patients who were anticipated to have a poor outcome due to older age or poor nutritional status.

The SGA was originally validated in surgical patients. After establishing its reliability and construct validity,^10^ its criterion (predictive) validity was established using the incidence of infections, use of antibiotics, and length of hospital stay, in surgical patients.^11^ Validation in other patient groups has mostly focused on the association of SGA with other measures^16^, ^40^ or against outcomes.^12^, ^13^, ^15^, ^16^ This audit confirms the ability of the SGA to predict a wider variety of outcomes in critically ill patients, including mortality, ICU length of stay, hospital readmission, and pressure injuries.

Several strengths and limitations are acknowledged for this retrospective audit. The major strengths of this study relate to its large sample size, heterogeneous study population, and consecutive sampling. To the authors' knowledge, this current study has analysed the largest patient dataset published to date. Importantly, these analyses adjusted for confounders and competing risk of mortality.

Age, sex and APACHE II score were found to have significant association with the outcomes, making it important to adjust for these influences. Selection bias arising from the competing risk of mortality is a perennial issue for analyses conducted in critically ill patients and the optimal strategy to address this remains controversial.^41^ Also controversial is the use of APACHE II score as a severity of illness measure. The purpose of APACHE II is to predict mortality.^32^ Although APACHE II score did have significant association with most of the outcomes considered in this study, not just mortality, it is a ‘soft’ measure, a composite of a large number of mortality risk predictors, and thus can be only a surrogate measure of illness severity. It therefore may not fully account for the confounding influence of other aspects of the patient's condition at ICU admission, other than when specifically analysing the relationship between SGA and mortality. For that analysis, it has been suggested that a more appropriate method would be to use the calculated hospital mortality risk (which uses the APACHE II score, weighted for the patient's ICU admission indication).^42^ This would certainly be more accurate when analysing hospital mortality as an outcome (although it might not have the same direct application to analyses for the other outcomes), but it should be noted that the sigmoid relationship between APACHE II score and calculated hospital mortality risk is quite linear except at the extremes of risk, so for the majority of patients it would make little difference to the analysis.

Other limitations of the study include, firstly, the retrospective nature of the audit and its reliance on the quality and accuracy of medical records documentation available. Due to the resource limitations of the study, it is likely that the mortality and readmission rates were underestimated as only those identified in the patient's electronic medical record were included. This would omit patients who were lost to follow‐up, or who received services or died outside the local health system where this was not recorded locally. Secondly, although the number of patient records was large, this audit was conducted only in a single centre, and not all patients in the ICU received a nutritional assessment on admission, reducing the generalisability of the results. The patients identified for nutritional assessment were either referred due to nutritional concerns or were starting nutrition support, and may represent a more nutritionally vulnerable subset of the ICU population with an over‐representation of malnourished patients; also there were many more surgical than medical patients, with a younger average age, and thus the associations found may not be representative of the broader patient population in intensive care. To address this, the validity of SGA in the ICU would ideally be established by way of a meta‐analysis of the existing diverse studies on this question. However, the conclusion of the current study is that protein‐energy malnutrition diagnosed by SGA is significantly associated with adverse clinical outcomes and this supports the predictive validity of SGA as a method of nutrition assessment in the critically ill.

## AUTHOR CONTRIBUTIONS

S.F. conceived the research, provided study oversight, and has primary responsibility for the final content. S.F. and N.B.W. drafted the manuscript. S.F., N.B.W., H.Y.C., S.T., and M.E.S. all contributed to the design of the research. All authors contributed to the collection, analysis and interpretation of the data, critically revised the manuscript, agree to be fully accountable for ensuring the integrity and accuracy of the work, and read and approved the final version of the manuscript.

## CONFLICT OF INTEREST

S. Ferrie is an Associate Editor of *Nutrition & Dietetics*. She was excluded from the peer review process and all decision‐making regarding this article. This manuscript has been managed throughout the review process by the Journal's Editor‐in‐Chief. The Journal operates a blinded peer review process and the peer reviewers for this manuscript were unaware of the authors of the manuscript. This process prevents authors who also hold an editorial role to influence the editorial decisions made. All other authors of this study have no conflicts to report.

## ETHICS STATEMENT

X19‐0059 & 2019/ETH00428 Indicators of Nutrition in Critical Illness and Severity of Illness scoring (the INCISI study) approved by the Royal Prince Alfred Hospital Ethics Review Committee.

## Data Availability

Data available on request, subject to ethics approval.
